# Concurrent associations between objective markers and subjective markers of aging with indicators of successful aging: An integrative approach

**DOI:** 10.1007/s10433-026-00920-1

**Published:** 2026-04-18

**Authors:** Fabio Selovin, M. Clara P. de Paula Couto, Markus Scholz, Ronny Baber, Steffi G. Riedel-Heller, Kerstin Wirkner, Samira Zeynalova, Klaus Rothermund

**Affiliations:** 1 Department of General Psychology II, Friedrich-Schiller-University Jena, Jena, Germany; 2 Insitute for Medical Informatics (IMISE), Statistics and Epidemiology, Leipzig University, Leipzig, Germany; 3 Leipzig Research Centre for Civilization Diseases (LIFE), Leipzig University, Leipzig, Germany; 4 Institute of Social Medicine, Occupational Health Und Public Health (ISAP), Leipzig University, Leipzig, Germany; 5 Institute of Laboratory Medicine, Clinical Chemistry, and Molecular Diagnostics, University Hospital Leipzig, Leipzig, Germany; 6 Leipzig Medical Biobank, Leipzig, Germany

**Keywords:** Markers of aging, Biological aging, Psychological aging, Successful aging, Subjective aging, Self-perceptions of aging

## Abstract

Cognitive decline, immune function, and inflammation are considered *objective markers of aging* (OMAs) and are commonly used to predict an individual’s aging success. We propose that subjective factors – such as a person’s beliefs and perceptions about their own age and aging – that have been shown to predict development in old age be defined as *subjective markers of aging* (SMAs). Although aging is a complex, multidimensional process, studies typically examine effects of OMAS and SMAs separately, rather than integrating both types of markers to improve predictions of successful aging. Our study aims to fill this gap by concurrently using both OMAs and SMAs to model the associations of different indicators of successful aging, including autonomous daily functioning, health-related quality of life, and life satisfaction. We assess OMAs through measures of visuo-motoric speed and executive function (Trail Making Test), inflammation (interleukin-6, c-reactive protein), and immune aging (neutrophil-to-lymphocyte ratio). In addition, we examine SMAs by evaluating subjective age and self-perceptions of aging. We used cross-sectional data from 456 older adults (*M*_Age_ = 74 years, 60–86 years) from the LIFE Adult Study to investigate the associations of OMAs and SMAs with successful aging. Regarding OMAs, cognitive functioning and inflammation were associated to daily activities and health-related quality of life, respectively, but were not related to life satisfaction. In contrast, both SMAs were associated to life satisfaction, and self-perceptions of aging were associated to health-related quality of life, but none of the SMAs were related to levels of daily functioning. Our results suggest that fundamental biological resources and psychological perceptions of aging are selectively associated with different facets of successful aging.

**Supplementary Information:**

The online version contains supplementary material available at 10.1007/s10433-026-00920-1.

Objective markers of aging (OMAs) are measurable indicators of the age-related deterioration in observable biological or physiological attributes of an organism in old age (e.g., immune function, grip strength, DNA methylation; Bao et al. 2023; Rothermund et al. 2023). A large number of studies have established OMAs as relevant predictors of aging-related outcomes, in particular focusing on the prediction of health/illness and longevity/mortality (Bao et al. 2023; Drewelies et al. 2022; Puzianowska-Kuźnicka et al. 2016). When evaluating the contribution of OMAs to aging well, it is important to recognize that – although grounded in biological processes – aging is a complex, multidimensional phenomenon (Baltes 1987) that is also influenced by individuals’ subjective beliefs and perceptions of their own age and aging. These subjective markers of aging (SMAs) are prognostic of well-being and health in later life, and facilitate adaptation to age-related health declines (Alonso Debreczeni and Bailey 2021; Westerhof et al. 2023; Wurm et al. 2008, 2017). Thus, SMAs should be considered alongside OMAs as important antecedents of an individual’s aging success (Sabatini et al. 2025). However, how OMAs and SMAs act in parallel and concurrently influence specific aspects of successful aging remains poorly understood (Gellert and Alonso-Perez 2024), since most studies tend to focus on either OMAs or SMAs in isolation (Hartanto et al. 2021; Schönstein et al. 2022).

Our study set out to relate different facets of successful aging, namely, functional ability, subjective health, and life satisfaction, with OMAs (cognitive function, immune function, and inflammation) as well as SMAs (subjective age and self-perceptions of one’s own aging) as independent variables in a cross-sectional sample. In the following sections, we will outline these success outcomes and introduce the OMAs and SMAs included in the study.

## Dimensions of successful aging

The study of success in aging debouched into a plethora of successful aging conceptions, that share the term *successful aging* but originated from vastly different understandings of the role of older adults and age in society (Wahl 2020). Most research considering success in aging follows either a pragmatic (functional) or an hedonic (reflecting evaluations of one’s life situation) conceptualization of success (Tesch-Romer et al. 2021). While pragmatic approaches consider preserving individual agency as the route to success, operationalizing success through indicators of high functioning (e.g., Rowe and Kahn 2015), hedonic approaches conceptualize success in aging as fundamentally subjective, proposing that being satisfied with one’s life and feeling subjectively healthy is the criterion for successful aging (e.g., Neugarten et al. 1961). So, while pragmatic approaches use functional outcomes as markers of successful aging, hedonic approaches emphasize the idiosyncratic nature of what is considered desirable in old age and focus on subjective evaluations of aging well.

To cover the spectrum from pragmatic to hedonic approaches to successful aging, we included different facets of successful aging as outcomes in our study. Autonomous daily functioning (IADL) was used as an indicator of functioning, whereas life satisfaction (LS) was included as an indicator of subjective evaluations of one’s life situation. In addition, we included health-related quality of life (hQoL) as a hybrid indicator. HQoL comprises objective bio-medical information (e.g., information of medical diagnosis, experiences of bodily sensations, current drug use, functional status) but also reflects evaluations of these indicators depending on personal goals and expectations of what health in old age looks like (Jylhä, 2009). The hQoL scale should thus be considered as a hybrid measure since it relates to functional aspects of health that are evaluated in relation to personal and societal standards.

## Objective markers of aging (OMAs)

### Inflammatory cytokines

Increases in chronological age are robustly associated with the emergence of a low grade chronic inflammatory state termed *inflamm-aging* (Franck et al. 2025). In contrast to acute inflammation, which contributes to organismic healing, the constant inflammatory background has been shown to be the driver in the development of age-related pathologies (e.g., arthritis, osteoporosis, arteriosclerosis), frailty, and disability in old age (Bao et al. 2023). The inflammatory status is indexed by blood-based inflammatory cytokines (e.g., Interleukin-6) that are secreted by cells of the innate immune system (e.g., neutrophils), and acute phase proteins (e.g., C-reactive Protein, CRP) secreted by the liver as an orchestrated response to inflammatory cytokines (Puzianowska-Kuźnicka et al. 2016).

### The balance of innate and adaptive immunity

**Fig. 1:**
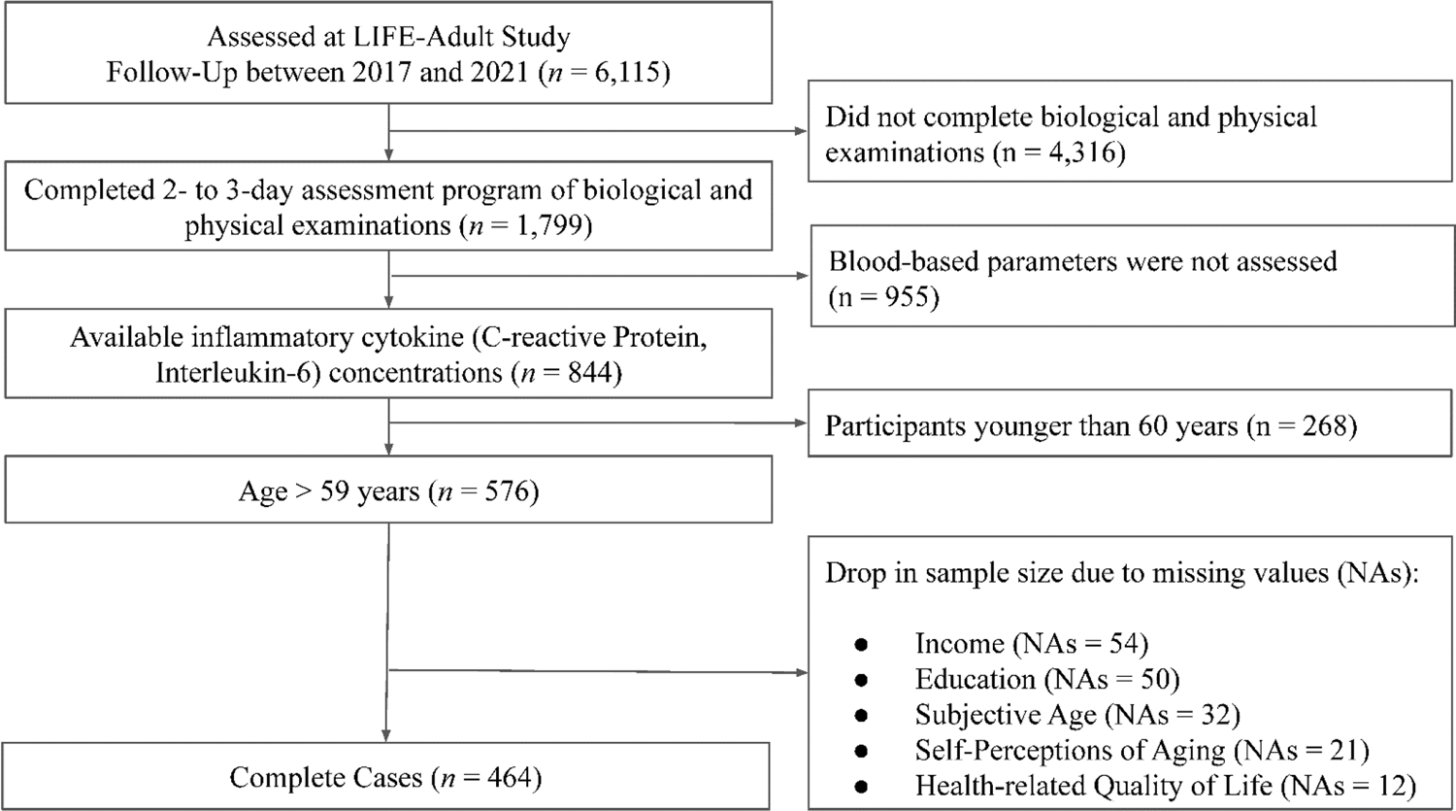
Flowchart of sample selection

Innate and adaptive immunity networks are dynamically remodelled with aging, resulting in an imbalance between inflammatory and anti-inflammatory networks (Bao et al. 2023). Specifically, aging is associated with heightened innate immunity and diminished adaptive immunity. This imbalance is reflected in the neutrophil-to-lymphocyte ratio (NL-R), calculated from the absolute number of neutrophils (innate immunity) and lymphocytes (adaptive immunity) in the blood. The NL-R has been shown to be associated with multimorbidity and frailty in old age (Pellegrino et al. 2024; Song et al. 2021) and due to its normative increase with chronological age can be considered an objective marker of immune system aging (Li et al. 2014) (Fig 1).

### Cognitive functioning

Due to morphological and functional changes in the cortical substrate that are normative for the aging process, cognitive performance declines progressively over the life-span (Murman 2015). Facets of cognitive performance that are sensitive to age-related decline are visuo-motoric speed and working memory (Lindenberger et al. 2001). This decline in cognitive functioning poses a risk for an individual’s ability to navigate everyday life independently (Johnson et al. 2007) and increases error proneness during high-risk daily activities (Toth et al. 2021).

However, it should be noted that cognitive performance comprises functioning in various cognitive domains (Harvey 2019). These domains are associated with specific brain networks and subnetworks that may compensate each other, rather than specific brain regions that deteriorate independently of one another (Drenth et al. 2023). Further, these different cognitive domains decline at varying rates (Heiskanen et al. 2024). This has important implications for empirical research and for our study. First, comprehensive cognitive assessments are necessary to account for the multidomain nature of cognitive performance and to support valid conclusions on cognitive performance. Second, specific cognitive domains may be related to specific successful aging outcomes, so statements about participants' cognitive performance can only be made in relation to a specific domain and the cognitive tests used.

## Subjective markers of aging

### Self-perceptions of aging and subjective age

Subjective views of aging are increasingly acknowledged as key psychological predictors of successful aging, due to their associations with various developmental outcomes in later life (Westerhof et al. 2023; Wurm et al. 2017). Research in this area suggests that subjective views of aging encompass distinct constructs (e.g., Shrira et al. 2022). Among these, self-perceptions of aging (SPA) and subjective age (SA) are prominent examples that assess how individuals experience and construe their own age and aging (Wurm et al. 2017). SPA refer to mental representations of one’s current and/or future age that motivate individuals to approach desired outcomes and avoid undesired ones as they age (Kornadt et al. 2015). SA or *felt age* indicates how old a person feels compared to their chronological age. A younger subjective age is associated to the activation of resources needed to attain desirable outcomes in old age (Alonso Debreczeni and Bailey 2021; Wienert et al. 2017). Numerous studies have shown the significant cross-sectional and longitudinal effects of SPA and SA on health, well-being, and longevity (Alonso Debreczeni and Bailey 2021; Westerhof et al. 2023; Wurm et al. 2017). These findings suggest that SMAs may be an important predictor for which individuals will be aging successfully and which are not.

## OMAs and successful aging

The associations of OMAs with indices of functional success (e.g., frailty, autonomous function) in older adults are consistently significant, with previous research showing that OMAs of cognitive functioning (Johnson et al. 2007; Toth et al. 2021), inflammation (Soysal et al. 2016), and immune deficit (Pellegrino et al. 2024) are cross-sectionally associated to better functional performance.

In contrast, cross-sectional findings on the association of inflammatory and immune function OMAs and subjective indicators of successful aging are inconsistent (de Vries et al. 2022). While some studies find significant bivariate associations showing that higher levels of inflammation (e.g., c-reactive protein, interleukin-6) and poorer immune function (e.g., white blood cell count) are linked to lower health-related quality of life (hQoL) or life satisfaction (LS; Friedman and Ryff 2012; Ironson et al. 2018), other studies find no such associations (Carpenter et al. 2012; Tait et al. 2019). This could be due to older adults anticipating a certain amount of decline and adjusting their expectation of what constitutes good *health* in old age (Nowakowski 2014). We consider this to be an important point. The psychological aging literature consistently shows that older adults have a strong tendency to rely on accommodative processes that help them accept losses and reinterpret problematic or critical life events in a more positive way (Brandtstädter & Rothermund 1994). Accommodative processes stabilize life satisfaction in old age even when action resources such as health or cognitive capacity are diminished (Rothermund and Brandtstädter 2003). Similarly, health problems are more likely to be positively reappraised and more easily accepted in old age (Aldwin et al. 2024) due to age-adjusted health expectations (Moser et al. 2013; Wurm et al. 2008), favourable downward comparisons (Heidrich and Ryff 1993), and the use of life management strategies of selection, optimization, and compensation (Jopp and Smith 2006). Taken together these psychological mechanisms can serve as buffers that make evaluative outcomes, such as life satisfaction and perceived quality of life, less dependent on fundamental resources like physical health, vitality, or cognitive functioning.

Cognitive OMAs like processing speed and verbal working memory have also been associated with LS cross-sectionally (Chen 2024; St. John and Montgomery 2010), indicating a positive relation between parameters of basic cognitive functioning and LS. In regard to the association of cognitive performance and hQoL, Park and Larson (2015) found that visuo-perceptual abilities and executive control were associated to hQoL in an older adult sample with chronic obstructive pulmonary disease. Brandão et al. (2020) found, in a healthy older sample, a significant positive relation between better cognitive function and higher QoL. However, none of the studies controlled for SMAs or *inflamm-aging*.

## SMAs and successful aging

People play an active role in guiding their aging (Rothermund et al. 2023). In young and middle adulthood, physiological resources (e.g., health) are abundant and can be freely invested to achieve desired goals or gain further resources. With increasing age physiological resources dwindle and as a result have to be managed and invested more cautiously to avoid further losses and still achieve gains in certain domains of life (Baltes 1987; Jopp and Smith 2006). To reduce losses and still achieve gains in old age the use of efficient resource management strategies (e.g., resource dependent goal selection, activation of priory unused resources) is crucial and leads to positive subjective health and well-being (Carpentieri et al. 2017).

Positive SPA and younger SA are personal resources that facilitate the individuals’ capacity to activate previously unused resources (e.g., self-efficacy beliefs, sense of control, optimism) and invest available resources (e.g., time) to achieve personally important goals (e.g., remaining healthy in old age; Klusmann et al. 2019; Kornadt et al. 2015). As a concrete example in the domain of health, Wurm et al. (2013) showed that negative SPA decreased the use of resource management strategies and in turn predicted lower subjective health and life satisfaction after a serious health event. On a related note, Wienert et al. (2017) showed that feeling younger is associated with a higher propensity of physical activity. The association was mediated by planning, indicating that a younger subjective age eases the efficient allocation of action resources to desired goals. Taken together the evidence suggests that SPA and SA promote subjective-related forms of success (e.g., life satisfaction, well-being) by aligning and activating available resources and the individuals’ goals for current gains.

## Concurrent relations of OMAs and SMAs for success in aging: the present study

The review of associations of OMAs and SMAs with success in aging suggests that these relationships are selective. OMAs reflect the availability of fundamental resources (e.g., health, vitality, cognitive functioning) needed to achieve predefined criteria for functional success. However, OMAs should be less strongly related to hedonic outcomes reflecting subjective evaluations of their life situation in old age, since accommodative processes in later life often make evaluative success in aging less dependent on the actual availability of fundamental resources (Rothermund and Brandtstädter 2003). SMAs on the other hand are psychological resource that promote correspondence between personal goals and available resources. They do so by maintaining a motivation to achieve desired outcomes or by adapting expectations regarding health and physical functioning in line with what is still feasible, thus shielding life satisfaction in old age against possible functional losses.

Based on these considerations, we predict that OMAs and SMAs show distinct relations with different indicators of successful aging. Specifically, we hypothesize that OMAs will be most strongly related to functional aspects of successful aging (instrumental activities of daily living, IADL) but not or to a lesser degree with hedonic indicators reflecting subjective evaluations of one’s life in old (life satisfaction, LS). The opposite pattern of predictions was made for SMAs, which were expected to be more strongly related to subjective, evaluative facets of successful aging (LS) but not functional performance (IADL). Both, OMAs and SMAs were assumed to be independently associated with health-related quality of life (hQoL), which is a hybrid construct comprising functional and hedonic (i.e., evaluative) aspects of successful aging.

By combining qualitatively different sources of data, our approach resembles the LOTS framework that has been proposed for personality research (Cervone and Pervin 2022; L = life record data, O = observer data, T = test data, S = self-report data). Our study specifically draws on objective biological and cognitive tests, as well as self-report measures of subjective health and quality of life to test facets of a comprehensive model of human aging (Rothermund et al. 2023).

## Methods

### Sample and procedure

Participants were drawn from the LIFE-Adult Study (Engel et al. 2023). The study was approved by the ethics board of the Medical Faculty of the University Leipzig (AZ 263–2009-14122009, 263/09-ff and 201/17-ek). For the baseline assessment (2011 – 2014), 10,000 participants aged between 18 – 86 years were randomly selected in an age- and sex-stratified manner from the population of Leipzig, Germany. Participants underwent a comprehensive assessment program. Approximately six years later (between 2017 and 2021), they were invited to participate in a follow-up. First, a set of paper-based, self-administered questionnaires was sent to all 10,000 baseline participants, of whom 6,115 responded and took part in the follow-up phase. From this group, a subsample of 1,799 participants underwent a 2- to 3-day assessment program of biological and physical examinations at the study center. The study variables used in the present analyses are drawn from this subsample. We used data from 844 individuals for which inflammatory cytokine concentrations were available. Due to our focus on investigating effects of OMAs and SMAs on indicators of successful aging, we restricted our analysis to a sample of individuals aged 60 years or older. Including younger participants would have produced effects not specific to old age. The final sample for whom data for all variables that were considered in the analyses were available consisted of *N* = 464 individuals (*M*_age_ = 74, *SD* = 6, 48% female, for detailed information about sample characteristic see Supplementary Table 1).

### Measures

#### Life satisfaction

We assessed life satisfaction with a single-item measure that has been employed in previous studies and has been shown to correlate highly with aggregate measures of life satisfaction that include multiple items assessing life satisfaction in different life-domains (e.g., Kornadt and Rothermund 2011; Voss et al. 2017). The item read, “*In general, how satisfied are you with your life*?” Participants indicated their life satisfaction on a 5-point Likert scale, ranging from 1 (*very dissatisfied*) to 5 (*very satisfied*). Higher values indicate higher levels of life satisfaction.

#### Health-related quality of life

Health-related quality of life (hQoL) was assessed using the SF-8 Health Survey (SF-8; Yiengprugsawan et al. 2020). The measure contains two psychometrically based physical (4 items) and mental health (4 items) summary measures, a measure of pain (1 item) and overall subjective health (1 item). Individuals indicate their subjective physical (e.g., “*In the past 4 weeks, how much have problems with your physical health restricted you in normal physical activities [walking, climbing stairs]*?”) and mental health (e.g., “*How much have mental problems [e.g. anxiety, depression or irritability] bothered you in the past 4 weeks*?”) on a 5-point Likert scale ranging from *“Not at all”* to *“Very strongly”,* and their experience of pain (e.g., “*How severe has your pain been in the past 4 weeks*?”) health on a 6-point scale ranging from *“Excellent”* to *“Very poor”.* We computed a composite score of health-related quality of life by averaging all items (α = 0.90). We reversed the item coding so that higher values indicate better hQoL.

#### Instrumental activities of daily living

Daily functioning and self-maintenance were assessed using the Instrumental Activities of Daily Living Scale (IADL; Graf 2008). The IADL is a clinical measure conceptualized to provide a screening of functional decline and capturing the need for further assessment or hospitalization. Individuals report their functioning in eight domains (ability to use the telephone, shopping, food preparation, housekeeping, laundry, mode of transportation, responsibility for own medications, ability to handle finances) by indicating whether they are either unable to perform (= 0) or able to perform (= 1). A summary score was computed by summing the responses across all domains, yielding a total score ranging from 0 (low function) to 8 (high function).

#### Inflammation biomarkers and immune function

Serum samples were processed by trained staff of the Leipzig Medical Biobank according to standard operating procedures and stored at -80 °C until analysis in the Institute of Laboratory Medicine at the University Hospital Leipzig. Systemic inflammation was indexed by two inflammation cytokines, Interleukin-6 and C-reactive Protein. High-sensitive CRP (mg/L) with a limit of detection of 0.15 mg/L and Interleukin-6 (pg/ml) with a lower limit of the assay of 1.5 pg/mL were measured according to standard procedure. Both measurements were log-transformed to normalize their distribution for statistical analysis. We also computed a composite inflammatory biomarker score (CII) by standardizing both measures and averaging the standardized scores (Hartanto et al. 2021). Immune function was indexed by the ratio of neutrophils to lymphocytes (Pellegrino et al. 2023). Since inflammatory cytokine concentrations and the neutrophils-to-lymphocyte ratio may increase drastically during acute viral and bacterial infections (Hartanto et al. 2021; Pellegrino et al. 2023) and we are interested in chronic inflammation and chronic disturbances in immune function in community dwelling older adults, we winsorized the inflammation index and NL-Ratio at the 95th percentile. Winsorizing is a statistical technique that limits biasing influences of extreme values in a dataset by replacing them with the nearest values within a specified percentile range (e.g., 95th percentile), reducing the influence of outliers and leading to more robust statistical results without removing data points (Wilcox 2012).

#### Cognitive indicators

The Trail Making Test (TMT; Bowie and Harvey 2006) was used to assess functioning in the cognitive domains of visuo-motoric abilities and working memory (Specka et al. 2022). The TMT is composed of two parts, A and B. In the TMT A, individuals are required to connect encircled numbers on a sheet of paper, in the TMT B individuals are required to alternate between numbers and letter (1-A-2-B). The test score is the amount of time in seconds an individual needs to connect numbers and letters, respectively. In case of an error, the examiner returns the individual to the last correct answer, prolonging test completion. It is presumed that Part A is a test of visual search and motor skills, while Part B has an added cognitive flexibility/executive function component. Since TMT B measures cognitive function in combination with visual search and motor skills, we regressed TMT B on TMT A test scores and used residual scores (TMT B-R) as a unique indicator of executive function (or cognitive flexibility) in our models. TMT A and TMT B-R levels that are higher than the 95th percentile may indicate other more extreme forms of cognitive or bodily impairments (e.g., partial blindness; Bowie and Harvey 2006). Since we are interested in community dwelling older adults, not in pathological aging, we winsorized TMT A and TMT B-R scores at the 95th percentile.

#### Self-perceptions of aging and subjective age

Participants were asked to report their subjective age in years (*In comparison to others: “How old do you currently feel”?* to which they responded, *“I feel as if I were _____ years old.”*). SA was computed as a proportional discrepancy score by subtracting chronological age from felt age, and then dividing by chronological age (Hartanto et al. 2021). A positive score indicates an older subjective age, while a negative score indicates a younger subjective age. In line with previous studies (Hartanto et al. 2021; Stephan et al. 2015) individuals with a discrepancy score higher than three standard deviations above and below the mean were winsorized. SPA were operationalized by the items: *“When I am older … I will be often sick/ I will have difficulties to stay mentally and physically fit/ I will be severely restricted in my daily life due to health problems”*. For analysis these items (α = 0.86) were averaged.

#### Covariates

In all models, gender (1 = male, 2 = female), education, and income were used as covariates. Education was indicated via self-report by selecting the highest achieved degree out of 13 ranked categories (1 = no school leaving qualification and no vocational qualification; 10 = general higher education entrance qualification and master’s degree or doctorate), we transformed the categories into years of education with a minimum of 18 and a maximum of 10 years. Monthly income was assessed via self-report by selecting one of 13 ranked categories ranging from a monthly net income of less than 800 € to more than 3000 €.

### Data analyses

The scope of the study was to assess the associative effect of established OMAs and SMAs on different outcomes related to success in old age, in a cross-sectional sample. IADL, hQoL, and LS were predicted by stepwise regression models. For each outcome, the analysis was carried out as follows: To control for any effects of the covariates, in the first step we entered chronological age, gender, education in years, and income (Covariate Model). In the second step (Model 1), we entered the OMA-related predictors, including the composite inflammation index (CII), the immune function indicator (NL-R) visuo-motoric deficits (TMT A) and executive function deficits (TMTB-R) as predictors. In the third step (Model 2), we entered the SMA-related predictors, including self-perceptions of aging (SPA) and subjective age (SA), the full model is thus akin to a simultaneous entry of all variables. We compared models by testing whether reduction in the residual sum of squares between Covariate Model, Model 1, and Model 2 was statistically significant. Model parameters were estimated using ordinary least square methods (OLS), all predictors and covariates were grand mean centered. We used R version 4.4.0, functions from the *stats* (R Core Team 2025) package for statistical analysis and *tidyverse* (Wickham et al. 2019) functions for data preparation. Model comparisons of the Covariate Model, Model 1 and Model 2 for IADL (Supplementary Table 2), hQoL (Supplementary Table 3), and LS (Supplementary Table 4) can be found in the Supplementary Material.

## Results

### Correlation results

Mean, standard deviations, and correlations between study variables can be found in Table 1 and Table 2. Neither IADL and LS nor IADL and hQoL correlated significantly. LS and hQoL showed a significant association (*r* = 0.22).

**Table 1:** Descriptive statistics of study variables

Variable	*M (SD)*	Min	Max	% (n)
Age	73.7 (5.7)	60	86	
Gender				
Male				52.6 (244)
Female				47.4 (220)
Income (EUR)				
Less than 800				2.8 (13)
800 to < 980				8.2 (38)
980 to < 1.100				6.5 (30)
1,100 to 1.200				11.0 (51)
> 1200 to < 1.333,33				5.8 (27)
1,333.33 to < 1.400				8.4 (39)
1,400 to < 1,533.33				8.8 (41)
1,533.33 to < 1,666.67				14.9 (69)
1,666.67 to < 1,866.67				6.7 (31)
1,866.67 to 2,000				7.3 (34)
> 2,000 to < 2,333.33				4.1 (19)
2,333.33 to < 3,000				7.1 (33)
3,000 or more				8.4 (39)
Education (in years)				
10				0.4 (2)
12				9.0 (42)
13				48.9 (227)
16				11.6 (54)
18				30.0 (139)
CII	0.0 (0.7)	− 1.2	1.8	
NL-R	2.7 (1.2)	0.1	5.5	
TMT A	43.5 (13.7)	20.0	74.0	
TMT B-R	0.0 (43.0)	− 89	156.0	
SA	−0.1 (0.1)	−0.5	0.2	
SPA	5.5 (1.5)	1.0	8.0	
IADL	7.8 (0.4)	4.0	8.0	
hQoL	4.2 (0.6)	1.8	5.25	
LS	4.0 (0.8)	1.0	5.0	

*Note. M* and *SD* are used to represent mean and standard deviation, respectively. CII: Composite Inflammatory Index. NL-R: Neutrophils to Lymphocyte Ratio (Immune function deficits). TMT A: Visuo-motor deficits. TMT B-R: Executive Function Deficit. IADL: Instrumental Activities of Daily Living (higher values reflect better functioning). hQoL: health-related Quality of Life. SA: Subjective Age (higher values indicate an older subjective age). SPA: Self-Perceptions of Aging (health), higher values indicate positive SPA. LS: Life Satisfaction

**Table 2:** Correlations of study variables

Variable	1	2	3	4	5	6	7	8	9	10	11	12
1. Age												
2. Gender [female]	−.13**											
3. Income	−.20**	−.11*										
4. Education (in years)	−.01	−.19**	.30**									
5. CII	.07	.00	−.08	−.12**								
6. NL-R	.21**	−.25**	−.10*	−.02	.21**							
7. TMT A	.35**	−.15**	−.10*	−.01	.09*	.13**						
8. TMT B-R	.18**	−.05	−.14**	−.18**	.12**	.04	.00					
9. SA	−.01	.04	−.02	.02	.04	.04	.10*	−.02				
10. SPA	−.12**	.13**	.10*	−.01	−.12**	−.12**	−.10*	−.08	−.23**			
11. IADL	−.16**	.20**	.02	−.03	−.02	−.08*	−.10*	−.16**	−.00	.06		
12. hQoL	−.18**	−.11**	.19**	.19**	−.15**	−.10*	−.12**	−.11**	−.16**	.32**	.03	
13. LS	−.07	−.00	.11*	−.00	−.02	.01	−.02	−.04	−.13**	.23**	.02	.22**

*Note.* CII: Composite Inflammatory Index. NL-R: Neutrophils to Lymphocyte Ratio (Immune function deficits). TMT A: Visuo-motor deficits. TMT B-R: Executive Function Deficit. IADL: Instrumental Activities of Daily Living (higher values reflect better functioning). hQoL: health-related Quality of Life. SA: Subjective Age (higher values indicate an older subjective age). SPA: Self-Perceptions of Aging (health). LS: Life Satisfaction. *p < .05, **p < 0.01, and ***p < 0.001

Covariates were selectively associated to the success in aging outcomes. More years of education was only significantly associated with better hQoL (*r* = 0.19), while higher income was significantly associated with better hQoL (*r* = 0.19) and with higher LS (*r* = 0.11). Older age was significantly correlated with lower IADL (*r* = -0.16) and with lower hQoL (*r* = −0.18) but not with LS (*r* = -0.07).

Overall, OMA-related variables were significantly correlated to hQoL (*rs* between −0.10 and −0.15) and IADL (*rs* between −0.02 and −0.16) but were not related to LS. Regarding SMA-related variables, overall, they were significantly correlated with hQoL (*r* = −0.16 and *r* = 0.32, for SA and SPA, respectively) and LS (*r* = −0.13 and *r* = 0.23, for SA and SPA, respectively), but not with IADL.

### Stepwise multiple linear regression results

Regarding the prediction of IADL (Table 3), we found that, in the Covariate Model, being female (*B* = 0.14, *p* < 0.001) and being younger (*B* = -0.01, *p* < 0.05) were associated to lower IADL. When OMAs were added to the model (Model 1), the effects of gender and age remained significant; however, the increase in explained variance was not significant (*F*(df) = 1.77(4), *p* = 0.13). In Model 1, higher executive function deficits (TMTB-R) significantly related to lower IADL (*B* = -0.001, *p* < 0.05); none of the other OMAs were significantly associated to IADL. Adding SMAs to the model (Model 2) did not significantly increase the explained variance (*F*(df) = 0.16(2), *p* = 0.84), and none of the SMAs were significantly associated to IADL.

**Table 3:** Multiple linear regression predicting success outcomes by OMAs and PMAs

	IADL						hQoL					Life Satisfaction						
	Model 1			Model 2			Model 1			Model 2			Model 1			Model 2		
*Predictors*	*b*	*t*	*p*	*b*	*t*	*p*	*b*	*t*	*p*	*b*	*t*	*p*	*b*	*t*	*p*	*b*	*t*	*p*
Intercept	−0.00	410.62	** < 0.001**	−0.00	409.87	** < 0.001**	0.00	148.03	** < 0.001**	0.00	155.61	** < 0.001**	0.00	109.68	** < 0.001**	0.00	112.22	** < 0.001**
Age	−0.10	−2.08	**0.038**	−0.10	−2.09	**0.037**	−0.14	−2.81	**0.005**	−0.14	−2.98	**0.003**	−0.04	−0.85	0.396	−0.05	−0.97	0.335
Gender	0.15	3.05	**0.002**	0.15	3.02	**0.003**	−0.11	−2.43	**0.015**	−0.13	−2.85	**0.005**	0.00	0.09	0.931	0.00	0.03	0.972
Income	0.01	0.20	0.842	0.01	0.16	0.873	0.09	1.84	0.067	0.06	1.41	0.158	0.11	2.21	**0.028**	0.09	1.91	0.057
Education	−0.04	−0.80	0.425	−0.04	−0.77	0.442	0.12	2.43	**0.015**	0.13	2.79	**0.005**	−0.04	−0.83	0.406	−0.03	−0.63	0.530
CII	−0.01	−0.28	0.778	−0.01	−0.24	0.810	−0.11	−2.41	**0.017**	−0.09	−1.93	0.054	−0.03	−0.56	0.577	−0.01	−0.20	0.841
NL-R	−0.02	−0.38	0.703	−0.02	−0.33	0.738	−0.06	−1.33	0.186	−0.04	−0.80	0.422	0.03	0.70	0.486	0.05	1.11	0.266
TMT A	−0.03	−0.63	0.530	−0.03	−0.57	0.570	−0.07	−1.44	0.150	−0.04	−0.98	0.328	−0.01	−0.15	0.881	0.02	0.32	0.746
TMT B-R	−0.12	−2.62	**0.009**	−0.12	−2.59	**0.010**	−0.04	−0.91	0.363	−0.03	−0.65	0.518	−0.06	−1.34	0.183	−0.06	−1.24	0.214
SA				−0.01	−0.29	0.771				−0.09	−1.95	0.051				−0.12	−2.50	**0.013**
SPA				0.02	0.38	0.706				0.27	6.13	** < 0.001**				0.16	3.43	**0.001**
n	464			464			464			464			464			464		
*R*^*2*^* / R*^*2*^*ad*	0.067 / 0.051			0.068 / 0.047			0.111 / 0.092			0.199 / 0.181			0.023 / 0.005			0.071 / 0.050		
Model Comp				Model 1 vs. Model 2						Model 1 vs. Model 2						Model 1 vs. Model 2		
*F*(df)				0.15(2)						**24.89(2)**						**11.80(2)**		

*Note.* CII: Composite Inflammatory Index. NL-R: Neutrophils to Lymphocyte Ratio (Immune function deficits). TMT A: Visuo-Motor Deficits. TMT B-R: Executive Function Deficit. SA: Subjective Age (higher values indicate older subjective age). SPA: Self-Perceptions of Aging (higher values indicate positive SPA). IADL: Instrumental Activities of Daily Living (higher values reflect better functioning). hQoL: health-related Quality of Life. *b*: standardized Beta, Significant estimates (*p* < .05) are bold. *R*^*2*^*:* R-squared. *R*^*2*^*ad*.: Adjusted R-squared

Considering hQoL (Table 3), we found that, in the Covariate Model, being younger (*B* = -0.02, *p* < 0.001), higher income (*B* = 0.04, *p* < 0.05), and more years of education (*B* = 0.04, *p* < 0.05) related to higher hQoL. When OMAs were added to the model (Model 1), explained variance increased significantly (*F*(df) = 3.74 (4), *p* < 0.05). Further, income was no longer significant; instead, education (*B* = 0.03, p < 0.05) and gender became significant (*B* = -0.15, *p* < 0.05). In Model 1, higher CII related to lower hQoL; no other OMA was significantly associated to hQoL. Adding SMAs to the model (Model 2) significantly increased explained variance (*F*(df) = 24.89(2), *p* < 0.001). In Model 2, younger age (*B* = -0.02, *p* < 0.05), male gender (*B* = -0.17, *p* < 0.05), and positive SPA (*B* = 0.11, *p* < 0.001) were significantly linked to hQoL. Lower CII (*B* = -0.07, *p* = 0.05) was marginally related to hQoL in Model 2.

Predicting LS (Table 3) using the Covariate Model showed that higher income (*B* = 0.05, *p* < 0.05) was significantly associated with higher LS. Adding OMAs to the model (Model 1) did not significantly increase explained variance (*F*(df) = 0.64(4), *p* = 0.63). Adding SMAs to the model (Model 2) did increase explained variance significantly (*F*(df) = 11.80(2), *p* < 0.001). In Model 2, younger SA (*B* = -1.00, *p* < 0.05) and positive SPA (*B* = 0.08, *p* < 0.001) were associated with higher LS.

## Discussion

The goal of the study was to examine the concurrent associations between objective markers of aging (OMAs) and subjective markers of aging (SMAs) for different forms of success in aging. Based on a cross-sectional sample of 464 community dwelling older adults we related different indicators of successful aging (autonomous daily functioning [IADL], health-related quality of life [hQoL], and life satisfaction [LS]) by OMAs (inflammation [CII], immune deficits [NL-R], visuo-motor deficits [TMT A], executive function deficit [TMTB-R]) and SMAs (subjective age [SA], self-perceptions of aging [SPA]). In line with assumptions derived from both biological and psychological aging literatures, we found different patterns of association for OMAs and SMAs in the correlative associations and in the concurrent relations with different facets of successful aging. Specifically, SMAs were associated with LS and hQoL, but not with IADL, while OMAs were associated with IADL and hQoL, but not with LS.

Our findings confirm and extend previous literature investigating effects of OMAs and SMAs on different dimensions of successful aging. Specifically, the null findings that were obtained for the relation between OMAs and hedonic, evaluative facets of successful aging replicate previous failures to find significant relations between OMAs and indicators of subjective well-being (Carpenter et al. 2012; Drewelies et al. 2022; Gow et al. 2005; Kim et al. 2021; Komura et al. 2023; Nowakowski 2014; Sutin et al. 2018; Tait et al. 2019; Vetter et al. 2022). Apparently, these more distal outcomes show no or only weak relations to biological and cognitive aging. This pattern may suggest that subjective evaluations are buffered against biological aging possibly by processes of psychological adaption, adjustment of evaluative standards, and reappraisal processes (Nowakowski 2014; Rothermund & Brandtstädter 2003; Wurm et al. 2008). However, we did not directly assess these processes in our study; the interpretation should thus be considered only as a speculative explanation that has to be tested in future studies.

In line with our conception of health-related quality of life (hQoL) as a hybrid indicator of successful aging, reflecting both functional (e.g., current drug use, information of medical diagnosis) and evaluative components (e.g., evaluation of medical information depending on personal goals and expectations), SMAs and OMAs (albeit marginally significant) showed, independent associations with this outcome. Although reappraisal processes may mitigate the impact of severe inflammation on life satisfaction, this mitigation may be more limited for health-related goals than for other life domains. Specifically, it has been found that personal health is more difficult to downgrade in importance (Brandtstädter and Rothermund 1994) due to health being a prerequisite for most other goals (Hobfoll 1989). Suggesting that evaluations of one’s subjective health and health-related quality of life may be less amendable to accommodative reinterpretations than other success outcomes (e.g., life satisfaction).

Replicating findings from the biomedical literature, we also found immune deficit or inflammation to be associated to lower levels of autonomous everyday functioning (IADL; see, e.g., Bao et al. 2023; Pellegrino et al. 2024). Extending this literature, we found a significant effect of executive function deficits on IADL. Shortcomings in cognitive functioning and the resulting increase in error proneness in everyday functioning are a primary concern for older adults, particularly in the areas of medication management, shopping, and health management (Toth et al. 2021). Individuals may be more aware of a reduction in executive function overall since actions slips in specific domains (e.g., medication management) can be costly. It is possible that specific activities of daily living are not performed due to perceptions that one’s cognitive status may lead to fatal outcomes, rather than due to fatigue or pain.

## Limitations

Though this study has several strengths (e.g., concurrent assessment of OMAs and SMAs), the following limitations should be noted. This study examines success in aging from a cross-sectional perspective. The associations found should be understood as a snapshot in time, considering static success based on the current availability of biological and cognitive (OMAs), as well as psychological (SMAs) resources.

Furthermore, our findings should be interpreted against the background that we did not control for the intake of pain medication or other compensatory measures. Our assessments of inflammation markers might directly be influenced by medication, since the levels of circulating inflammatory cytokines are directly influenced by the use of non-steroidal anti-inflammatory drugs (NSAIDs; e.g., ibuprofen, diclofenac, aspirin, etc.), which are among the most commonly used over-the-counter drugs worldwide (Pilotto et al. 2010). Due to these compensatory effects, our assessments might thus reflect an overly optimistic picture of actual levels of biological aging. Importantly, effects of OMAs on outcomes might be weakened due to the alleviating effects that these medications can have on pain perception and mobility. However, the significant association of hQoL with CII suggests that inflammatory signals were strong enough to affect perceived health but not daily functioning.

Another limitation is the range of our indicators of successful aging. A variety of indicators (e.g., self-acceptance, wisdom, generativity) and conceptions of successful aging have been discussed in the literature (Wahl 2020) of which our approach is but a selection. Our selection was driven by (a) the availability of measures, and (b) the aim to capture both functional and hedonic indicators of successful aging, which are the most prominent dimensions of successful aging. Still, we acknowledge the pluralism in conceptions of successful aging and expect that the concept of “aging well” will be a subject of ongoing discussions due to diverse and sometimes contradictory value systems on what a “good life” (pleasure vs. longevity) in old age is and on the question of who is responsible (individual vs. society) for a “good life” in old age.

A further limitation of the present study concerns the use of the Instrumental Activities of Daily Living (IADL) scale in a sample of community-dwelling older adults. The IADL captures dimensions of basic everyday functioning, which are highly relevant for living autonomously and independently, which is of high importance for older adults (e.g., de Paula Couto et al. 2023). However, the IADL scale is primarily designed to detect functional decline in samples were reductions in functionality are expected and is therefore less sensitive to subtle variations in higher levels of functioning. Consequently, the IADL may have underestimated variability in functional outcomes among relatively high-functioning individuals.

We assessed Life Satisfaction with a global single-item measure, which has been shown to perform similarly to the Satisfaction with Life Scale in terms of validity and reliability (e.g., Cheung and Lucas 2014). Future studies should consider comparing how OMAs and SMAs impact domain-specific Life Satisfaction.

Lastly, our study consisted of a sample of community-dwelling (i.e., non-institutionalized, healthy) older adults, and thus did not include test batteries that allow for a comprehensive assessment of disease-based and pathological impairments of cognitive functioning (e.g., MCI or dementia). Although these diseases will also result in reduced performance on the TMT, this test is insufficient for a comprehensive assessment of pathological impairments in cognitive functioning. Therefore, our results should be interpreted with caution. Future studies should consider cognitive screening measures that account for a variety of cognitive domains that allow for a screening for dementia and pathological forms of cognitive impairment (e.g., Mini-Mental State Examination, MMSE (Folstein et al. 1975); Montreal Cognitive Assessment, MoCA (Nasreddine et al. 2005); or General Practitioner Assessment of Cognition, GPCog (Wojtowicz and Larner 2015). The reported findings might thus underestimate effects of cognitive functioning on indicators of successful aging, and future studies should include a more comprehensive assessment of cognitive functioning.

## Conclusion

Our findings indicate that OMAs and SMAs have distinct relations with functional and hedonic indicators of successful aging. While OMAs were primarily related to functional indicators (autonomous daily functioning), SMAs were primarily related to hedonic outcomes that reflect subjective evaluations of life in old age. By introducing a dimensional approach that distinguishes between objective (biological/cognitive) and subjective (psychological) markers of aging, on the one hand, and between functional (IADL) and hedonic (hQoL, LS) indicators of successful aging, on the other hand, we have achieved a taxonomy of antecedents and outcomes that allows for specific predictions regarding the strength of the relations. In a nutshell, our findings suggest that OMAs relate to functional outcomes, whereas SMAs mostly relate hedonic outcomes. This dimensional approach allows an integration of the previous literature, providing explanations for seemingly heterogenous and contradictory findings relating markers of aging to indicators of successful aging.

## Supplementary Information

Below is the link to the electronic supplementary material.

Supplementary file1 (DOCX 34 KB)

## Data Availability

Data were drawn from the LIFE-Adult-Study, access to the study's original data is possible per formal project agreement via the LIFE data portal (https://ldp.life.uni-leipzig.de/). The studies’ hypotheses and analytical plan were not preregistered.
