# Supplementary material for: Concurrent associations between objective markers and subjective markers of aging with indicators of successful aging: An integrative approach
Source: Eur J Ageing. 2026 Apr 18;23(1):18. doi: 10.1007/s10433-026-00920-1 (PMC13096469; doi:10.1007/s10433-026-00920-1)
Supplement: Supplementary file 1 — Supplementary file1 (DOCX 34 KB) [file 10433_2026_920_MOESM1_ESM.docx]

**Supplementary Material**

| **Supplementary Table 1** | | | |
| --- | --- | --- | --- |
| *Descriptive Statistics of Study Variables* | | | |
|  | | Analytical Sample w/o missing values (n = 464) |  |
| Variables | | Statistics | Count of missing values before exclusion |
| Age, M (SD; range) | | 73.7 (5.7; 60 – 86) | 0 |
| Gender, n (%) | |  | 0 |
|  | Woman | 220 (47.4) |  |
|  | Man | 244 (52.6) |  |
| Educational Attainment^c^, n (%) | |  | 50 |
|  | Secondary school or PSC qualification and no vocational qualification | 2 (00.4) |  |
|  | No school leaving certificate or secondary school leaving certificate and apprenticeship | 45 (09.7) |  |
|  | Secondary school or POS qualification and apprenticeship | 166 (35.8) |  |
|  | Technical college entrance qualification or high school diploma and no vocational qualification | 3 (00.6) |  |
|  | Secondary school leaving certificate and technical college degree | 4 (00.8) |  |
|  | Secondary school or POS qualification and technical college degree | 38 (08.2) |  |
|  | Technical college entrance qualification and vocational training/apprenticeship/technical college | 21 (04.5) |  |
|  | Secondary school leaving certificate and university degree | 1 (00.2) |  |
|  | Secondary school or POS qualification and university degree | 12 (02.6) |  |
|  | Technical college entrance qualification and bachelor's degree or diploma from a technical college | 33 (07.1) |  |
|  | Technical college entrance qualification and master's degree/diploma, doctorate | 139 (30.0) |  |
| Income, n (%) | |  | 54 |
|  | Less than 800 EUR | 13 (02.8) |  |
|  | 800 to < 980 EUR | 38 (08.2) |  |
|  | 980 to < 1100 EUR | 30 (06.5) |  |
|  | 1100 to 1200 EUR | 51 (11.0) |  |
|  | > 1200 to < 1333,33 EUR | 27 (05.8) |  |
|  | 1333,33 to < 1400 EUR | 39 (08.4) |  |
|  | 1400 to < 1533,33 EUR | 41 (08.8) |  |
|  | 1533,33 to < 1666,67 EUR | 69 (14.9) |  |
|  | 1666,67 to < 1866,67 | 31 (06.7) |  |
|  | 1866,67 to 2000 EUR | 34 (07.3) |  |
|  | > 2000 to < 2333,33 EUR | 19 (04.0) |  |
|  | 2333,33 to < 3000 EUR | 33 (07.1) |  |
|  | More than 3000 EUR | 39 (08.4) |  |
| CII, M (SD; range) | | **0.05 (0.74; -1.21 – 1.86)**  0.09 (0.87; -1.21 – 4.75) |  |
|  | Interleukin – 6, M (SD; range) | **4.01 (1.04; 3.50 – 7.11)**  4.29 (2.39; 3.50 – 29.30) | 3 |
|  | C-reactive Protein, M (SD; range) | **2.07 (1.79; 0.22 – 6.91)**  2.42 (4.28; 0.22 – 72.64) | 3 |
| NL-R, M (SD; range) | | **2.71 (1.17; 0.11 – 5.53)**  2.79 (1.47; 0.11 – 15.17) |  |
|  | Neutrophils, M (SD; range) | **3.72 (1.16; 1.25 – 6.17)**  3.76 (1.25; 1.25 – 10.03) | 3 |
|  | Lymphocyte, M (SD; range) | **1.48 (0.43; 0.24 – 2.35)**  1.55 (1.36; 0.24– 28.97) | 3 |
| Trail Making Test A, M (SD; range) | | **43.10 (13.54; 20.0 – 74.00)**  44.21 (16.69; 20.0 – 180.0) | 1 |
| Trail Making Test B residuals, M (SD; range) | | **-0.9 (40.7; -89.5 – 156.0)** | 0 |
| Subjective Age, M (SD; range) | | -0.08^a^ (0.09; -0.53 – 0.20)^a^  -0.08 (0.10; -1.00 – 0.27) | 32 |
| Future Self-Views, M (SD; range) | | 5.56 (1.53; 1 – 8) | 21 |
| IADL, M (SD; range) | | 7.88 (0.42; 4 – 8) | 1 |
| hQoL, M (SD; range) | | 4.24 (0.65; 1.88 – 5.25) | 12 |
| Life Satisfaction, M (SD; range) | | 4.02 (0.79; 1.0 – 5.0) | 7 |
| *Note.* M: mean, SD: standard deviation. Bold values are based on Winsorized (upper 95^th^ percentile) variables. ^a^: values based on variables Winsorized 3 SD below and above the mean. ^c^: any participants had ‘school qualification and no vocational qualification’ or ‘secondary school leaving certificate and no vocational qualification’. PSC: polytechnic secondary school. M: mean, SD: standard deviation. CII: composite inflammatory index, IADL: instrumental activities of daily living, hQoL: health-related quality of life. | | | |

| **Supplementary Table 2** | | | | | | | | | |
| --- | --- | --- | --- | --- | --- | --- | --- | --- | --- |
| *Multiple Linear Regression Predicting Instrumental Activities of Daily Living (IADL) by OMAs and SMAs* | | | | | | | | | |
|  | IADL | | | | | | | | |
|  | **Covariate Model** | | | **Model 1** | | | **Model 2** | | |
| *Predictors* | *β* | *t* | *p* | *β* | *t* | *p* | *β* | *t* | *p* |
| (Intercept) | -0.00 | 409.23 | **<0.001** | -0.00 | 410.62 | **<0.001** | -0.00 | 409.86 | **<0.001** |
| Age | -0.14 | -2.92 | **0.004** | -0.11 | -2.11 | **0.035** | -0.11 | -2.12 | **0.035** |
| Gender | 0.17 | 3.53 | **<0.001** | 0.15 | 3.09 | **0.002** | 0.15 | 3.06 | **0.002** |
| Income | 0.01 | 0.21 | 0.830 | 0.00 | 0.07 | 0.948 | 0.00 | 0.03 | 0.976 |
| Education | 0.02 | 0.31 | 0.758 | -0.01 | -0.23 | 0.814 | -0.01 | -0.23 | 0.822 |
| CII |  |  |  | -0.01 | -0.23 | 0.820 | -0.01 | -0.19 | 0.853 |
| NL-R |  |  |  | -0.02 | -0.38 | 0.707 | -0.02 | -0.33 | 0.744 |
| TMT A |  |  |  | -0.03 | -0.63 | 0.531 | -0.03 | -0.56 | 0.575 |
| TMT B-R |  |  |  | -0.12 | -2.54 | **0.011** | -0.12 | -2.52 | **0.012** |
| SA |  |  |  |  |  |  | -0.02 | -0.33 | 0.744 |
| SPA |  |  |  |  |  |  | 0.02 | 0.39 | 0.700 |
| R^2^ / R^2^adj. | 0.052 / 0.043 | | | 0.066 / 0.050 | | | 0.067 / 0.046 | | |
| Model Comp.  F(df) |  |  |  | Covariate Model vs. Model 1  1.77(4) | | | Model 1 vs. Model 2  0.16(2) | | |
| *Note*. CII: Composite Inflammatory Index. NL-R: Neutrophils to Lymphocyte Ratio (Immune function deficits). TMT A: Visuo-Motor Deficits. TMT B-R: Executive Function Deficit. SA: Subjective Age Bias. SPA: Self-Perceptions of Aging. R^2^ad.: Adjusted R-squared. Significant estimates (p < .05) are bold. | | | | | | | | | |

| **Supplementary Table 3** | | | | | | | | | |
| --- | --- | --- | --- | --- | --- | --- | --- | --- | --- |
| *Multiple Linear Regression Predicting health-related Quality of Life (hQoL) by OMAs and SMAs* | | | | | | | | | |
|  | hQoL | | | | | | | | |
|  | **Covariate Model** | | | **Model 1** | | | **Model 2** | | |
| *Predictors* | *β* | *t* | *p* | *β* | *t* | *p* | *β* | *t* | *p* |
| (Intercept) | 0.00 | 145.99 | **<0.001** | 0.00 | 147.51 | **<0.001** | 0.00 | 154.79 | **<0.001** |
| Age | -0.17 | -3.74 | **<0.001** | -0.14 | -2.78 | **0.006** | -0.14 | -2.92 | **0.004** |
| Gender | -0.08 | -1.80 | 0.072 | -0.12 | -2.42 | **0.016** | -0.13 | -2.86 | **0.004** |
| Income | 0.11 | 2.13 | **0.033** | 0.09 | 1.86 | 0.063 | 0.07 | 1.52 | 0.130 |
| Education | 0.11 | 2.23 | **0.026** | 0.08 | 1.64 | 0.102 | 0.08 | 1.72 | 0.086 |
| CII |  |  |  | -0.11 | -2.47 | **0.014** | -0.09 | -2.03 | **0.043** |
| NL-R |  |  |  | -0.06 | -1.33 | 0.184 | -0.04 | -0.82 | 0.412 |
| TMT A |  |  |  | -0.07 | -1.41 | 0.159 | -0.04 | -0.96 | 0.335 |
| TMT B-R |  |  |  | -0.04 | -0.91 | 0.363 | -0.03 | -0.68 | 0.499 |
| SA |  |  |  |  |  |  | -0.08 | -1.86 | 0.064 |
| SPA |  |  |  |  |  |  | 0.27 | 6.05 | **<0.001** |
| R^2^ / R^2^adj. | 0.078 / 0.070 | | | 0.105 / 0.089 | | | 0.191 / 0.173 | | |
| Model Comp.  F(df) |  |  |  | Covariate Model vs. Model 1  **3.74(4)** | | | Model 1 vs. Model 2  **24.01(2)** | | |
| *Note*. CII: Composite Inflammatory Index. NL-R: Neutrophils to Lymphocyte Ratio (Immune function deficits). TMT A: Visuo-Motor Deficits. TMT B-R: Executive Function Deficit. SA: Subjective Age Bias. SPA: Self-Perceptions of Aging. R^2^ad.: Adjusted R-squared. Significant estimates (p < .05) are bold. | | | | | | | | | |

| **Supplementary Table 4** | | | | | | | | | |
| --- | --- | --- | --- | --- | --- | --- | --- | --- | --- |
| *Multiple Linear Regression Predicting Life Satisfaction (LS) by OMAs and SMAs* | | | | | | | | | |
|  | LS | | | | | | | | |
|  | **Covariate Model** | | | **Model 1** | | | **Model 2** | | |
| *Predictors* | *β* | *t* | *p* | *β* | *t* | *p* | *β* | *t* | *p* |
| (Intercept) | 0.00 | 109.78 | **<0.001** | 0.00 | 109.60 | **<0.001** | 0.00 | 112.17 | **<0.001** |
| Age | -0.05 | -1.12 | 0.263 | -0.05 | -0.89 | 0.376 | -0.05 | -0.99 | 0.321 |
| Gender | 0.01 | 0.11 | 0.910 | 0.01 | 0.16 | 0.869 | 0.00 | 0.09 | 0.925 |
| Income | 0.10 | 2.04 | **0.042** | 0.10 | 2.01 | **0.045** | 0.09 | 1.76 | 0.079 |
| Education | 0.00 | 0.06 | 0.952 | -0.01 | -0.23 | 0.815 | -0.01 | -0.18 | 0.858 |
| CII |  |  |  | -0.02 | -0.50 | 0.618 | -0.01 | -0.16 | 0.876 |
| NL-R |  |  |  | 0.03 | 0.70 | 0.483 | 0.05 | 1.12 | 0.264 |
| TMT A |  |  |  | -0.01 | -0.15 | 0.882 | 0.02 | 0.33 | 0.742 |
| TMT B-R |  |  |  | -0.06 | -1.26 | 0.207 | -0.06 | -1.19 | 0.235 |
| SA |  |  |  | 0.00 | 109.60 | **<0.001** | -0.12 | -2.53 | **0.012** |
| SPA |  |  |  |  |  |  | 0.16 | 3.44 | **0.001** |
| R^2^ / R^2^adj. | 0.018 / 0.005 | | | 0.021 / 0.004 | | | 0.070 / 0.049 | | |
| Model Comp.  F(df) |  |  |  | Covariate Model vs. Model 1  0.64(4) | | | Model 1 vs. Model 2  **11.80(2)** | | |
| *Note*. CII: Composite Inflammatory Index. NL-R: Neutrophils to Lymphocyte Ratio (Immune function deficits). TMT A: Visuo-Motor Deficits. TMT B-R: Executive Function Deficit. SA: Subjective Age Bias. SPA: Self-Perceptions of Aging. R^2^ad.: Adjusted R-squared. Significant estimates (p < .05) are bold. | | | | | | | | | |
